# Early Effect Markers and Exposure Determinants of Metalworking Fluids Among Metal Industry Workers: Protocol for a Field Study

**DOI:** 10.2196/13744

**Published:** 2019-08-02

**Authors:** Nancy B Hopf, Eve Bourgkard, Valérie Demange, Sébastien Hulo, Jean-Jacques Sauvain, Ronan Levilly, Fanny Jeandel, Alain Robert, Yves Guichard, Jacques André Pralong, Nathalie Chérot-Kornobis, Jean-Louis Edmé, Pascal Wild

**Affiliations:** 1 Department of Occupational and Environmental Health Center for Primary Care and Public Health (Unisanté) University Lausanne Lausanne Switzerland; 2 Department of Epidemiology National Research and Safety Institute (INRS) Vandoeuvre cedex France; 3 IMPECS- EA 4483 Department of Occupational Health Lille University Hospital Lille France; 4 Process Engineering Department National Research and Safety Institute (INRS) Vandoeuvre cedex France; 5 Toxicology and Biometrology Department National Research and Safety Institute (INRS) Vandoeuvre cedex France; 6 National Research and Safety Institute (INRS) Vandoeuvre cedex France

**Keywords:** metalworking fluid, oxidative stress, exposure biomarkers, early effect biomarkers, genotoxic effects, occupational epidemiology

## Abstract

**Background:**

Exposure to aerosols from metalworking fluids (MWF) has previously been related to a series of adverse health outcomes (eg, cancer, respiratory diseases). Our present epidemiological study focuses on occupational exposures to MWF and a panel of exposure and effect biomarkers. We hypothesize that these health outcomes are caused by particle exposure that generates oxidative stress, leading to airway inflammation and ultimately to chronic respiratory diseases. We aimed to assess whether MWF exposure, in particular as characterized by its oxidative potential, is associated with biomarkers of oxidative stress and inflammation as well as genotoxic effects.

**Objective:**

The ultimate goal is to develop exposure reduction strategies based on exposure determinants that best predict MWF-related health outcomes. The following relationships will be explored: (1) exposure determinants and measured exposure; (2) occupational exposure and preclinical and clinical effect markers; (3) exposure biomarkers and biomarkers of effect in both exhaled breath condensate and urine; and (4) biomarkers of effect, genotoxic effects and respiratory symptoms.

**Methods:**

At least 90 workers from France and Switzerland (30 controls, 30 exposed to straight MWF and 30 to aqueous MWF) were followed over three consecutive days after a nonexposed period of at least two days. The exposure assessment is based on MWF, metal, aldehyde, and ultrafine particle number concentrations, as well as the intrinsic oxidative potential of aerosols. Furthermore, exposure biomarkers such as metals, metabolites of polycyclic aromatic hydrocarbons and nitrosamine are measured in exhaled breath condensate and urine. Oxidative stress biomarkers (malondialdehyde, 8-isoprostane, 8-hydroxy-2’-deoxyguanosine, nitrates, and nitrites) and exhaled nitric oxide, an airway inflammation marker, are repeatedly measured in exhaled breath condensate and urine. Genotoxic effects are assessed using the buccal micronucleus cytome assay. The statistical analyses will include modelling exposure as a function of exposure determinants, modelling the evolution of the biomarkers of exposure and effect as a function of the measured exposure, and modelling respiratory symptoms and genotoxic effects as a function of the assessed long-term exposure.

**Results:**

Data collection, which occurred from January 2018 until June 2019, included 20 companies. At the date of writing, the study included 100 subjects and 29 nonoccupationally exposed controls.

**Conclusions:**

This study is unique as it comprises human biological samples, questionnaires, and MWF exposure measurement. The biomarkers collected in our study are all noninvasive and are useful in monitoring MWF exposed workers. The aim is to develop preventative strategies based on exposure determinants related to health outcomes.

**International Registered Report Identifier (IRRID):**

DERR1-10.2196/13744

## Introduction

### Metalworking Fluids and Their Aerosols

Metalworking fluids (MWF) are used to lubricate and cool tools and the workpiece, as well as flush away metal chips during machining, cutting, grinding, and drilling of metals in many manufacturing processes, from small parts in the watch-making industry to large parts in the automotive or steel industries. MWF are classified into two main families [1]: (1) Straight MWF that is mineral oil containing no water; and (2) Aqueous MWF that regroup so-called soluble oils and semisynthetic fluids according to the amount of mineral oils emulsified in water, as well as synthetic fluids that contain no mineral oil. Depending on their type and use, MWF may contain lubricity, antimisting or antiwear additives, corrosion inhibitors and biocides, as well as perfumes or coloring agents.

Other substances potentially present in the aerosols from used MWF are the result of thermal degradation or contamination of the machined metal parts. Thus, MWF may contain: (1) polycyclic aromatic hydrocarbons (PAHs) due to lack of an initial refining stage or due to thermal degradation; (2) nitrosamines, present in aqueous MWF as by-products of the reaction between secondary amines and nitrite; or (3) microorganisms like bacteria or mycobacteria that may be growing in tanks containing aqueous MWF.

The physical process of metalworking generates a complex MWF aerosol consisting of droplets (the oil mist), which may contain solid particles (eg, metals), and a vapor phase (air or organic vapors). This vapor phase is the result of the evaporation of volatile or semivolatile constituents from the MWF in contact with the hot cutting zone. These aerosols can reach the workers’ breathing zone and may remain in suspension for several hours [1]. The size distribution of the MWF aerosols is highly variable (median aerodynamic diameters ranging from 1.8-17 µm) and may contain ultrafine particles (aerodynamic diameter <0.1µm) [2]. MWF aerosols may be inhaled or enter the body through skin contact. Exposure from soiled clothing and ingestion (hand to mouth contamination) are also possible. Consequently, assessing occupational exposure to MWF aerosols has many challenges.

### Health Effects From Exposure to Metalworking Fluids

Historically, exposure to poorly refined straight oil-mists has been related to cancer of the skin and the scrotum [3]. More recently, there is growing evidence of a relationship between exposure to straight MWF and bladder cancer [4,5]. As early as the 1990s, exposure to oil-mist was related to acute bronchial hyperresponsiveness, occupational asthma, hypersensitivity pneumonitis, ventilatory impairments and respiratory symptoms [6-9]. Recently, a causal model was applied [10] to explore the quantitative relationship between exposure to MWF aerosol and chronic obstructive pulmonary disease (COPD).

### Presumed Physiopathological Mechanisms

**Figure 1:**
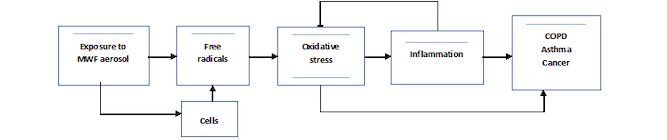
Presumed physio-pathological mechanism adapted from Ayres et al. (2008).

Inflammation is hypothesized to be an important process that explains some of the observed health outcomes. Indeed, in vivo chronic exposure to rather high concentrations of different semisynthetic MWF have resulted in inflammation in rat and mice lungs [11]. In addition, signs of oxidative stress have also been reported on the skin of vitamin E deficient mice exposed to MWF [12]. Based on this, Figure 1 (adapted from Ayres et al [13] who presented this mechanism for environmental particulate exposure) summarizes the presumed physiopathological mechanism. Given the multiplicity of health outcomes that have been related to oil-mist exposure, we assumed that the main, common mechanism of these effects was oxidative stress. Briefly, the exposure to the MWF aerosols generates free radicals in the lungs of both exogenous and endogenous origin and thereby causes oxidative stress. This oxidative stress induces inflammation, which ultimately increases the oxidative stress via a feedback mechanism. Thus, this chronic inflammation eventually leads to chronic adverse health effects.

## Methods

### The Study Protocol

#### The Study Objectives

The study protocol very closely followed the mechanistic pathway outlined in Figure 1 by proposing an epidemiological field study in both the Swiss micromechanical industry and the French metal industry. It assessed the exposure to MWF approximating the biological effect dose, the oxidative stress, inflammation, genotoxic cellular modifications, and the early nonspecific effect biomarkers that might be on the pathways that lead to chronic respiratory diseases.

**Figure 2:**
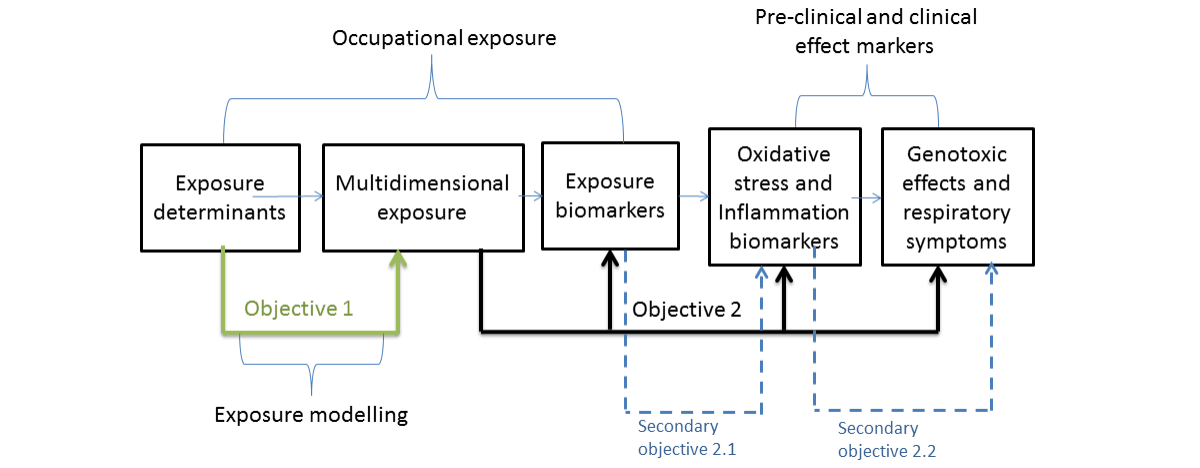
Schematic presentation of the study objectives.

The ultimate objective is to develop exposure reduction strategies based on exposure determinants that best predict MWF-related health outcomes. This ultimate objective is broken down into several partial objectives summarized in Figure 2. The first primary objective of the study, though, was to establish relationships between the exposure determinants and the exposure measurements. The second primary objective was to establish relationships between occupational exposure, especially oxidative potential, and preclinical and clinical effect markers. There were also two secondary objectives, which included establishing relationships between exposure biomarkers and biomarkers of effect in exhaled breath condensate (EBC) and Urine, as well as establishing relationships between biomarkers of effect, genotoxic effects and respiratory symptoms.

#### Protocol of the Epidemiological Field Study

A three day long longitudinal study of exposed versus nonexposed workers, after a nonexposed period of at least two days, was conducted in Swiss and French companies. At least 30 workers were exposed to straight oil and 30 workers were exposed to water-based MWF. Nonexposed workers from the participating companies will be included, at a ratio of 2 exposed for 1 nonexposed.

The exclusion criteria for this study were: (1) known chronic or acute respiratory diseases; and (2) known exposure to particulate substances with a potential effect on oxidative stress.

**Figure 3:**
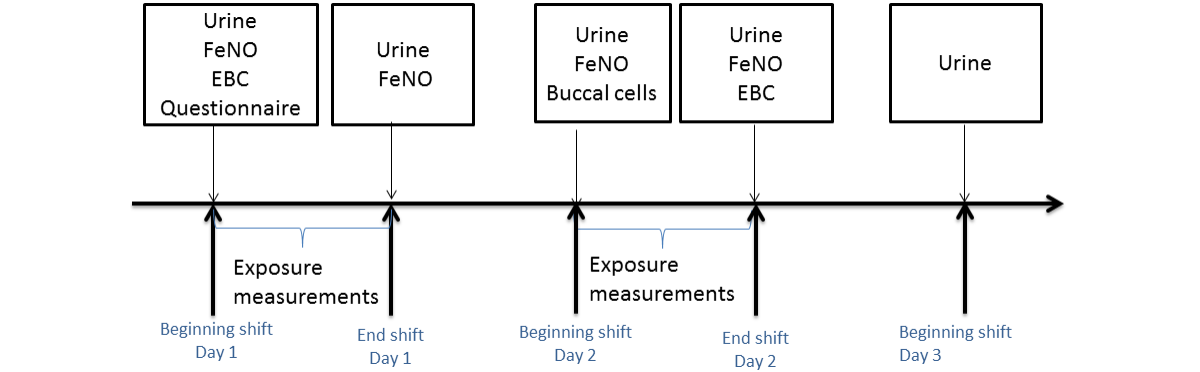
Schematic presentation of the field study protocol. EBC: exhaled breath condensate; FeNO: fractional exhaled nitric oxide.

The data collection is summarized in Figure 3. It will consist of repeated characterization of airborne exposure during two consecutive days and multiple collections of EBC, urine and fractional exhaled nitric oxide (FeNO) in parallel. Buccal cells will be collected once per participant. A questionnaire that explores respiratory symptoms, sociodemographic factors (smoking and clinical history, age, etc), the present job, tasks, personal protective equipment, and the participant’s job history will also be applied.

EBC will be collected at the beginning of the shift on Day 1 and at the end of Day 2. Urine samples will be collected at the beginning of shift on Days 1, 2 and 3 as well as at the end of the shift on Days 1 and 2. The FeNO will be measured at the beginning and end of shift on Days 1 and 2. The exposure measurements will be obtained over the shifts of Day 1 and 2.

In addition to these data, the occupational hygienists will record workers’ tasks and the corresponding exposure determinants. Finally, samples of both used and new MWF will be obtained.

### Exposure and Health Outcome Assessment

Table 1 summarizes the different outcomes measured during the field study.

**Table 1:** Outcomes measured in the field study.

Outcome type		Measured outcome	Method
Exposure determinants		Determinants	Questionnaire
**Exposure measurements**			
	Personal sampling	Standard gravimetric exposure Organic carbon Measurement of ultrafine particle numbers	Metropol M-282 Thermal degradation method Real time instrument
	Stationary sampling	Standard gravimetric exposure Oxidative potential Aldehydes Metals Organic carbon NO_2_^– a^, NO_3_^– b^ Measurements of ultrafine particle numbers	Metropol M-282 Ferrous oxidation-xylenol orange method Metropol M4 and M66 Metropol M122 Thermal degradation method Ion chromatography Real time instrument
	Monitoring of new and used MWF^c^	Oxidative potential Metals Organic carbon Benzo(a)Pyrene	Ferrous oxidation-xylenol orange assay ICP-AES^d^ Thermal degradation method HPLC-Fluo^e^
**Biomarkers of exposure**			
	In exhaled breath condensate	NO_2_^–^/NO_3_^–^ Metals	Ion chromatography ICP-MS^f^
	In urine	NDELA^g^ 1-OHP^i^, 3-OHBaP^j^ (PAH^k^ metabolites) Metals	HPLC-MS/MS^h^ HPLC-Fluo ICP-MS
**Biomarkers of effect**			
	In exhaled breath condensate	MDA^l^, 8-isoprostane (markers of lipid peroxidation) 8-OHdG^m^ (marker of DNA oxidation) Formate, NO_2_^–^/NO_3_^–^ (proposed markers of nitrosative stress)	HPLC-MS/MS HPLC-MS/MS Ion chromatography
	In urine	MDA, 8-isoprostane (markers of lipid peroxidation) 8-OHdG (marker of DNA oxidation)	HPLC-MS/MS HPLC-MS/MS
**Other effect markers**		Micronuclei in buccal cells (marker of genotoxicity) Fractional Exhaled Nitric Oxide (marker of eosinophilic inflammation) Respiratory symptoms	Buccal micronucleus cytome assay Direct-reading instrument Standardized questionnaire

^a^NO_2_^–^: nitrite

^b^NO_3_^–^: nitrate

^c^MWF: metalworking fluids

^d^ICP-AES: inductively coupled plasma atomic emission spectroscopy

^e^HPLC-Fluo: high-performance liquid chromatography with fluorescence detection

^f^ICP-MS: inductively coupled plasma mass spectrometer

^g^NDELA: N-nitrosodiethanolamine

^h^HPLC-MS/MS: high-performance liquid chromatography coupled to tandem mass spectrometry

^i^1-OHP: 1-hydroxypyrene

^j^3-OHBaP: 3-hydroxybenzo(a)pyrene

^k^PAH: polycyclic aromatic hydrocarbons

^l^MDA: malondialdehyde

^m^8-OHdG: 8-Oxo-2'-deoxyguanosine

### Exposure Determinants

Exposure determinants are factors within the workplace that contribute to increasing or reducing exposure concentrations [14]. A questionnaire has been developed based on the literature [1,15] and will be used by the occupational hygienists during the field study.

### Multidimensional Features of Exposure

#### Respirable Aerosol Exposure

The respirable particulate MWF mass fraction from personal and stationary sampling will be determined following a reference gravimetric method (INRS Metropol M-282). As a complement to these measurements, the organic carbon content of the aerosol collected on quartz filters will be determined using a specifically modified thermal degradation method [16]. The MWF concentration of the volatile organic fraction will be sampled using a sorbent tube and analyzed chemically. The combination of these measurements will be used to evaluate the overall airborne MWF.

#### Oxidative Potential

Using the particulate oxidative potential as an additional metric for evaluating possible toxic effects is a relatively new concept and has rarely been used in occupational health studies. In our study, the oxidative potential of the MWF themselves (new and used) for the respirable fraction of the aerosol, as well as for the gaseous phase, will be quantified. The oxidative potential method used will be based on the ferrous oxidation-xylenol orange method [17]. Briefly, MWF was sampled with a teflon filter for the particulate phase followed by a XAD-2 sorbent tube for the gaseous phase. An acidic solution of iron(II) (Fe^2+^) with xylenol orange as the indicator and sorbitol as a catalyst (ferrous ion oxidation [FOX] solution) was prepared. Oxidation of Fe^2+^ to iron(III) (Fe^3+^) was followed by calorimetry using a spectrometer. Increasing hydrogen peroxide (H_2_ O_2_) (aq) (0-10 uM) concentrations were used for calibration for the filters and in dimethyl sulfoxide for the XAD-2 sorbent. The teflon filter with the particulate MWF was punched and dropped into the FOX solution, vortexed (1 min), and analyzed. The XAD-2 sorbent was desorbed with dichloromethane, evaporated, resuspended in dimethyl sulfoxide, and analyzed.

#### Components in the Metalworking Fluid Aerosol

The metal content (eg, iron (Fe), copper (Cu), aluminum (Al), zinc (Zn), manganese (Mn), antimony (Sb), cobalt (Co), nickel (Ni), chromium (Cr), molybdenum (Mo), vanadium (V) and titanium (Ti)) of the aerosol will be measured using stationary samples with cellulose acetate filters, which will be mineralized and analyzed using inductively coupled plasma atomic emission spectroscopy (ICP-AES). Finally, aldehyde concentrations including acetaldehyde and formaldehyde will be quantified.

#### Real Time Measurement of Ultrafine Particles

Exposure to ultrafine particles might be related to adverse health effects, and ultrafine particles are present in MWF aerosol [2]. Particle number concentration of ultrafine particles will be measured using a real time particle counter (DiscMini) in the 10-500 nm range. The particle size distribution between 0.25-32 µm will be determined with an optical particle counter (Grimm Optical Particle Counter 1.109, 31 channels).

### Exposure Biomarkers

EBC is an emerging technique that is simple, noninvasive and allows scientists to study processes in the lungs [18]. The metal concentrations in EBC (eg, Fe, Mn, Cr, Ni, Cu, Zn) will be determined with an inductively coupled plasma mass spectrometer (ICP-MS) using the analytical Totalquant technique with external calibration [19,20].

We will measure the following urinary exposure biomarkers: metals (27 metals), a nitrosamine (N-nitrosodiethanolamine [NDELA]) and two PAH metabolites (1-hydroxypyrene [1-OHP], a metabolite of pyrene, and 3-hydroxybenzo(a)pyrene [3-OHBaP], a metabolite of benzo(a)pyrene). Metals will be analyzed using ICP-MS [21], whereas the PAH metabolites will be analyzed using high-performance liquid chromatography with fluorescence detection (HPLC-Fluo) [22]. Finally, a more sensitive method for analyzing urinary NDELA will be developed based on high-performance liquid chromatography coupled to tandem mass spectrometry (HPLC-MS/MS).

### Inflammation and Oxidative or Nitrosative Stress Biomarkers

#### Inflammation Biomarker

Nitric oxide is a biomarker of bronchial inflammation often used [23] in an occupational context, and it is related to other inflammation markers like nonspecific bronchial hyperresponsiveness, a presumed precursor of asthma [24]. FeNO is a noninvasive and easy method of measuring nitric oxide, with standardized commercial devices (Niox Vero) available.

#### Oxidative or Nitrosative Stress Biomarkers in Exhaled Breath Condensate

Following Basu [25]:

Isoprostanes, mainly 8-iso-PGF2αand 8-iso-PGE2, possess potent biologic effects in a number of biologic systems, and thus they may also serve as pathologic mediators of oxidant stress through their vasoconstrictive and inflammatory properties.

8-iso-prostaglandin F_2α_ (8-iso-PGF_2α_) is a marker of a pathway in the free radical lipid peroxidation mechanism, and therefore fits our presumed mechanism shown in Figure 1. Malondialdehyde (MDA) is another indirect marker of lipid peroxidation, although it is considered less specific. Concentrations of 8-Oxo-2'-deoxyguanosine (8-OHdG) are considered a trace of a repairing/excretion mechanism for oxidized guanine and are considered a measure of whole-body oxidative stress. 8-isoprostane and 8-OHdG will be analyzed following the publication by Syslova [26] using HPLC-MS/MS. The same technique will be used for MDA analysis after a derivatization step.

Nitrites and nitrates have previously been identified as pollutants in aqueous MWF and are markers of nitrosative stress [27,28]. These two anions will be measured in EBC by ion chromatography.

#### Oxidative Stress Biomarkers in Urine

8-isoprostane, MDA and 8-OHdG will also be analyzed in the urine using HPLC-MS/MS [29,30].

### Genotoxic Effects and Respiratory Symptoms

#### Markers of Genotoxicity

Genotoxicity from MWF aerosol exposure will be assessed using the buccal micronucleus cytome assay [31]. The presence of micronuclei in buccal cells is considered a sign of damage to the DNA and of chromosomal instability. Buccal cells will be harvested from each participant. Using a microscope with white and fluorescent light, the cells will be stained using cytoplasmic and DNA staining and then 2000 cells will be scored for the presence of micronuclei or nuclear buds. A number of occupational exposures have given rise to excess numbers of micronuclei [32].

#### Respiratory Symptoms

Symptoms of chronic bronchitis and asthma-like conditions will be explored using the standardized Epidemiological study on the Genetics and Environment of Asthma, bronchial hyperresponsiveness and atopy (EGEA) questionnaire [33]. In addition, the questionnaire will ask for cutaneous symptoms (eczema or eczema-like symptoms).

### Statistical Methods and Power

The statistical analysis will follow the presumed causal model (Figure 2) and will be performed using the Stata (StataCorp LP, College station, Texas) statistical software. The first analysis will model the exposure measurements as a function of the determinants using linear models, after suitable transformations (logarithmic) if necessary. The exposure biomarkers in EBC and urine will be modelled as a function of the exposure measurements as well as possibly the exposure determinants. These analyses will focus on the within-shift evolution of these markers and will be based on mixed models. Measurements below the limits of detection will be included using specific models (random effect Tobit or Bayesian models) [34]. The biomarkers of effect and the FeNO will be analyzed as a function of the exposure measurements and determinants using similar statistical models. Again, the focus will be on the within-shift evolution but also on the evolution over the three days. The effects from the circadian cycle will be controlled by the simultaneous modelling of the nonexposed subjects. The prevalence of symptoms and the frequency of micronuclei will be analyzed using logistic regression as a function of the exposed or nonexposed status and the chronic occupational exposure that will be estimated from the job history and the exposure determinants. Note that in the analysis of the frequency of micronuclei, the number of collected cells per subject will be included as an offset and that this analysis will account for a possible overdispersion using a negative binomial regression. Possible confounders such as smoking, age, sex and diet will be accounted for in the different statistical analyses.

The study size was determined based on the longitudinal evolution of FeNO and the comparison of the frequency of micronuclei in two exposure groups. According to Bohadana et al [35], a 10% increase of FeNO between 2 measurements corresponds to an 80% power at a 5% significance level with a sample of 30 subjects.

With respect to micronuclei, assuming a 0.74% [36] baseline prevalence among controls, we have an 80% power at a 5% significance level with two samples of 30 subjects. This allows us to detect a 2.2 rate ratio between an exposed and a nonexposed group assuming a 1.5 between-subject geometric SD.

The rationale behind choosing the number of controls in a 1:2 ratio to exposed workers is to get equal sized exposure groups between the aqueous exposed works, the straight oil exposed workers, and the controls.

### Study Organization

The present study is carried out by a consortium of three organizations: (1) Team 1, the research team EA 4483 from the IMPact de l’Environnement Chimique sur la Santé humaine (IMPECS; Impact of the chemical environment on human health) of the University of Lille in France; (2) Team 2, Unisanté, the department of occupational and environmental health from the University of Lausanne in Switzerland; and finally (3) Team 3, the Institut National de Recherche et de Sécurité (INRS; French National Institute for Research and Safety) in France.

The study is coordinated by the INRS. A Consortium Agreement specifying the legal, financial and scientific framework of the cooperation regarding the present study was signed by the three organizations on March 22, 2017.

Two parallel groups of scientists in Switzerland and France with common operational procedures carry out the on-site data collection. To minimize laboratory bias, most of the samples are dispatched to and analyzed by one laboratory only. All organic biomarkers in urine and EBC, as well as the micronuclei frequency in buccal cells, will be analyzed by team 3. The metals in EBC will be analyzed [14] by team 1. Team 2 will characterize MWF aerosol for its oxidative potential, organic carbon, nitrate, nitrite and aldehyde content as well as the formate concentration in EBC. Both Team 2 and 3 will determine the particulate exposure.

All electronic documents related to the study are deposited in a structured, encrypted extranet located on a server of team 3. The access to this extranet is strictly restricted to the study team members by an individual password. Measurement data from each lab will be deposited by the laboratories generating them. All deposited data will be anonymous, and the rules for generating the identification codes are defined by an operational procedure. The data on the extranet will be saved daily and backups will be kept in a separate building from the server. The deposited data will be organized and prepared for data analysis in the three months after collection by the same data manager, who will provide regular feedback on the data collection.

### Ethics and Data Dissemination

This study was approved by the ethical committee of the canton Vaud, Switzerland (Commission cantonale d’éthique de la recherche sur l’être humain CER-VD) on June 13, 2017, project number 2017-00630, and by France (Comité de Protection des Personnes Sud-Est) on May 17, 2017, project number 17-02-EE-VD/OXIGENOCOM.

Several scientific, peer-reviewed publications are planned. The first ones will correspond to the preparatory phases (eg, a scientific article on the determination of biomarkers in EBC, a paper on automatic versus human-based counting of micronuclei in buccal cells). The other planned papers will respectively correspond to the objectives described in the beginning of this paper.

The ultimate objective is to develop relevant exposure reduction strategies. The results will be published in a nonscientific format and will be accessible by environmental health and safety professionals.

Depending on the corresponding legal authorizations, parts or all of the data will be deposited on a public data repository after the final publications.

## Results

Our study is organized into three partially overlapping periods. The first period started October 2016 and ended December 2017 and was dedicated to: (1) obtaining the ethics committees’ consents; (2) writing and agreeing on operational procedures; (3) setting up a study-specific data extranet; and (4) validating analytical laboratory procedures.

The second period started with a pilot study conducted from February 12, 2018 to Feb 14, 2018. The subsequent debriefing led to minor adjustments of the daily organization of the data collection. The data collection will end in June 2019. At the time of writing, 100 subjects from 17 companies have been included, comprising 29 control subjects. An additional three companies will be included.

The third period consists of laboratory analysis, data management, and data analysis. Written feedback will be provided to participants and companies related to exposure. Finally, a scientific report will be sent to the funding agency in April 2020, and results will be published in peer-reviewed journals.

## Discussion

A strength of this project is its hypothesis driven and multidisciplinary approach. First, the diversity of biomarkers will shed some light on the physiopathological mechanisms. Second, the extensive exposure assessment by occupational hygienists will help in characterizing components that have a short-term effect on the measured biomarkers. Finally, recording exposure determinants will help with focusing the future exposure preventions so that they have the greatest potential impact on workers’ health.

One of the most interesting parameters is the oxidative potential of the MWF aerosol. We hypothesize that oxidative potential is a measure capturing the overall oxidative stress generated by the aerosol and would thus be independent of the inert (or nonreactive) constituents such as hydrocarbons. Thus, one of the most interesting relationships to be explored is between oxidative potential and the biomarkers of effect, in particular the biomarkers of oxidative stress. In the words of Dr Ken Donaldson [13]:

The measurement of the oxidative potential of ambient particles would represent a more refined metric, bringing it closer to the Biological Effect Dose with anticipated improvements in risk management and better associations with adverse health effects in epidemiological studies.

Indeed, diverse approaches to assessing occupational exposure give us the tools to characterize what might be the biological effect dose.

Another strength is the noninvasive biomarker collection approach. Respiratory symptoms and urinary biomarkers, and to a lesser extent FeNO, are often used in occupational epidemiology. EBC and micronuclei frequencies in buccal cells are not routinely used yet; however, these biomarkers have shown great promise in earlier work from our research group [28,29,32,37,38]. Thus, the markers developed in this project could potentially be used in routine monitoring of exposed workers.

An important aspect of our study is that the relationships we want to study are in two different time-frames. The relationships between the exposure measurements and the evolution of the biomarkers, be they measured in exhaled air, its condensate or in urine, reflect short-term relationships. The second time frame we consider is chronic. Neither the respiratory symptoms nor the micronuclei frequency are assumed to vary in the 2 days of data collection. These outcomes are therefore obtained only once and reflect long-term effects. To some extent, we could also consider the effect markers measured Monday morning as possible long-term markers. However, when analyzing these outcomes as a function of exposure, we have to consider long-term exposure. The latter is necessarily less precise than the measured exposure because it has to be assessed using the jobs’ histories and tasks recorded in the questionnaire.

The constraints of our study protocol entailed a very intensive field data collection from no more than 4 study subjects, with 4 researchers and technicians present in the companies over 4 days. This is the drawback of the very complete exposure assessment, but also of the required participation time from each worker, and finally of the required 2-day unexposed period before inclusion. The total sample size is thus limited. To be able to identify the exposure measurements that are closest to the biological effective dose, it will be necessary to include workers, and hence companies, with varied exposure characteristics both qualitatively and in terms of exposure concentrations. Bacterial contamination and endotoxin measurements were not included due to limited resources. Nevertheless, our protocol reflects a multidisciplinary approach and allows small or very small companies to be included. Consequently, the likelihood of having high exposure levels is greater compared to large companies, which are the ones usually explored in epidemiological studies.

Thus, the possible lack of power induced by the relatively small number of included subjects will be compensated for by the large exposure variance when including both highly exposed and less exposed workers. It is noteworthy that the repeated measure design (five measurements per subject for urine and two for EBC) will contribute to increase the power of detecting short-term effects.

For outcomes collected only once reflecting long-term effects (respiratory symptoms or micronuclei), the power will correspondingly be lower, especially as the chronic exposure estimate will be less precise and possibly affected by exposure misclassification. As shown previously, one would need an odds ratio greater than 2 to be able to detect such effects.

Related to statistical power is the issue of multiplicity. Indeed, the number of outcomes will be quite large, so a number of statistical models will be fitted to more or less the same data. Thus, with the exception of the central hypothesis relating oxidative potential measurements to oxidative stress, it will certainly be safe to consider some analyses as exploratory or to assign some multiplicity correction to any *P* value.

### Conclusion

This study is unique, as it comprises human biological samples, questionnaires, and MWF exposure measurement. The aim is to develop preventative strategies based on exposure determinants related to health outcomes. To achieve this goal, this integrative multidisciplinary approach quantifies the relationships between exposure determinants, exposure measurements, biomarkers of exposure, biomarkers of effect, and early effect outcomes.

## Abbreviations

1-OHP: 1-hydroxypyrene
8-iso-PGE2: 8-isoprostaglandin E2
8-iso-PGF2α: 8-iso-prostaglandin F2α
8-OHdG: 8-Oxo-2'-deoxyguanosine
Al: aluminum
Co: cobalt
COPD: chronic obstructive pulmonary disease
Cr: chromium
Cu: copper
EBC: exhaled breath condensate
EGEA: epidemiological study on the genetics and environment of Asthma, bronchial hyperresponsiveness and atopy
Fe: iron
Fe2+: iron(II)
Fe3+: iron(III)
FeNO: fractional exhaled nitric oxide
FOX: ferrous ion oxidation
H2O2: hydrogen peroxide
ICP-AES: inductively coupled plasma atomic emission spectroscopy
ICP-MS: inductively coupled plasma mass spectrometer
IMPECS: IMPact de l’Environnement Chimique sur la Santé humaine
INRS: institut national de recherche et de sécurité
MDA: malondialdehyde
Mn: manganese
Mo: molybdenum
MWF: metalworking fluids
NDELA: N-nitrosodiethanolamine
Ni: nickel
PAH: polycyclic aromatic hydrocarbons
Sb: antimony
Ti: titanium
V: vanadium
Zn: zinc

## References

[1] Lillienberg L, Burdorf A, Mathiasson L, Thörneby L (2008). Exposure to metalworking fluid aerosols and determinants of exposure. Ann Occup Hyg.

[2] Heitbrink WA, Evans DE, Peters TM, Slavin TJ (2007). Characterization and mapping of very fine particles in an engine machining and assembly facility. J Occup Environ Hyg.

[3] (1984). Polynuclear Aromatic Hydrocarbons, Part 2, Carbon Blacks, Mineral Oils (Lubricant Base Oils and Derived Products) and some Nitroarenes. IARC Monographs on the Evaluation of the Carcinogenic Risk of chemicals to humans.

[4] Colt JS, Friesen MC, Stewart PA, Donguk P, Johnson A, Schwenn M, Karagas MR, Armenti K, Waddell R, Verrill C, Ward MH, Freeman LEB, Moore LE, Koutros S, Baris D, Silverman DT (2014). A case-control study of occupational exposure to metalworking fluids and bladder cancer risk among men. Occup Environ Med.

[5] Colin Régis, Grzebyk Michel, Wild Pascal, Hédelin Guy, Bourgkard Eve (2018). Bladder cancer and occupational exposure to metalworking fluid mist: a counter-matched case-control study in French steel-producing factories. Occup Environ Med.

[6] Kennedy S, Greaves I, Kriebel D, Eisen E, Smith T, Woskie S (1989). Acute pulmonary responses among automobile workers exposed to aerosols of machining fluids. Am J Ind Med.

[7] Ameille Jacques, Wild Pascal, Choudat Dominique, Ohl Gérard, Vaucouleur Jean-François, Chanut Jean, Brochard Patrick (1995). Respiratory symptoms, ventilatory impairment, and bronchial reactivity in oil mist-exposed automobile workers. Am J Ind Med.

[8] Wild Pascal, Ameille Jacques (1997). Bronchial reactivity in oil-mist exposed automobile workers revisited. Am J Ind Med.

[9] Eisen E, Bardin J, Gore R, Woskie S, Hallock M, Monson R (2001). Exposure-response models based on extended follow-up of a cohort mortality study in the automobile industry. Scand J Work Environ Health.

[10] Picciotto S, Chevrier J, Balmes J, Eisen E (2014). Hypothetical interventions to limit metalworking fluid exposures and their effects on COPD mortality: G-estimation within a public health framework. Epidemiology.

[11] Ryan K, Cesta M, Herbert R, Brix A, Cora M, Witt K, Kissling Grace, Morgan Daniel L (2017). Comparative pulmonary toxicity of inhaled metalworking fluids in rats and mice. Toxicol Ind Health.

[12] Shvedova A, Kisin E, Murray A, Smith C, Castranova V, Kommineni C (2002). Enhanced oxidative stress in the skin of vitamin E deficient mice exposed to semisynthetic metal working fluids. Toxicology.

[13] Ayres J, Borm P, Cassee F, Castranova V, Donaldson K, Ghio A, Harrison Roy M, Hider Robert, Kelly Frank, Kooter Ingeborg M, Marano Francelyne, Maynard Robert L, Mudway Ian, Nel Andre, Sioutas Constantinos, Smith Steve, Baeza-Squiban Armelle, Cho Art, Duggan Sean, Froines John (2008). Evaluating the toxicity of airborne particulate matter and nanoparticles by measuring oxidative stress potential--a workshop report and consensus statement. Inhal Toxicol.

[14] Burstyn I, Teschke K (1999). Studying the determinants of exposure: a review of methods. Am Ind Hyg Assoc J.

[15] Park Donguk, Stewart Patrica A, Coble Joseph B (2009). Determinants of exposure to metalworking fluid aerosols: a literature review and analysis of reported measurements. Ann Occup Hyg.

[16] Chow J, Watson J, Chen L, Chang M, Robinson N, Trimble D, Kohl Steven (2007). The IMPROVE_A temperature protocol for thermal/optical carbon analysis: maintaining consistency with a long-term database. J Air Waste Manag Assoc.

[17] Laulagnet A, Sauvain J, Concha-Lozano N, Riediker M, Suárez G (2015). Sensitive Photonic System to Measure Oxidative Potential of Airborne Nanoparticles and ROS Levels in Exhaled Air. Procedia Engineering.

[18] Mutlu G, Garey K, Robbins R, Danziger L, Rubinstein I (2001). Collection and analysis of exhaled breath condensate in humans. Am J Respir Crit Care Med.

[19] Becker S (2007). Inorganic Mass Spectrometry: Principles and Applications.

[20] Hulo S, Radauceanu A, Chérot-Kornobis Nathalie, Howsam M, Vacchina V, De Broucker Virginie, Rousset Davy, Grzebyk Michel, Dziurla Mathieu, Sobaszek Annie, Edme Jean-Louis (2016). Beryllium in exhaled breath condensate as a biomarker of occupational exposure in a primary aluminum production plant. Int J Hyg Environ Health.

[21] Goullé Jean-Pierre, Mahieu L, Castermant J, Neveu N, Bonneau L, Lainé Gilbert, Bouige Daniel, Lacroix Christian (2005). Metal and metalloid multi-elementary ICP-MS validation in whole blood, plasma, urine and hair. Reference values. Forensic Sci Int.

[22] Simon P, Lafontaine M, Delsaut P, Morele Y, Nicot T (2000). Trace determination of urinary 3-hydroxybenzo[a]pyrene by automated column-switching high-performance liquid chromatography. J Chromatogr B Biomed Sci Appl.

[23] Chérot-Kornobis Nathalie, Hulo Sébastien, de Broucker Virginie, Hassoun Sidi, Lepage Nadège, Edmé Jean Louis, Sobaszek Annie (2012). Induced sputum, exhaled NO, and breath condensate in occupational medicine. J Occup Environ Med.

[24] Demange Valérie, Bohadana Abraham, Massin Nicole, Wild Pascal (2009). Exhaled nitric oxide and airway hyperresponsiveness in workers: a preliminary study in lifeguards. BMC Pulm Med.

[25] Basu S (2008). F2-isoprostanes in human health and diseases: from molecular mechanisms to clinical implications. Antioxid Redox Signal.

[26] Syslová K, Kačer P, Kuzma M, Pankrácová A, Fenclová Z, Vlčková S, Lebedová J, Pelclová D (2010). LC-ESI-MS/MS method for oxidative stress multimarker screening in the exhaled breath condensate of asbestosis/silicosis patients. J Breath Res.

[27] Horváth I, Hunt J, Barnes P, Alving K, Antczak A, Baraldi E, Becher G, van Beurden W J C, Corradi M, Dekhuijzen R, Dweik R A, Dwyer T, Effros R, Erzurum S, Gaston B, Gessner C, Greening A, Ho L P, Hohlfeld J, Jöbsis Q, Laskowski D, Loukides S, Marlin D, Montuschi P, Olin A C, Redington A E, Reinhold P, van Rensen E L J, Rubinstein I, Silkoff P, Toren K, Vass G, Vogelberg C, Wirtz H, ATS/ERS Task Force on Exhaled Breath Condensate (2005). Exhaled breath condensate: methodological recommendations and unresolved questions. Eur Respir J.

[28] Chérot-Kornobis Nathalie, Hulo Sébastien, Edmé Jean-Louis, de Broucker Virginie, Matran R, Sobaszek Annie (2011). Analysis of nitrogen oxides (NOx) in the exhaled breath condensate (EBC) of subjects with asthma as a complement to exhaled nitric oxide (FeNO) measurements: a cross-sectional study. BMC Res Notes.

[29] Sauvain Jean-Jacques, Setyan Ari, Wild Pascal, Tacchini Philippe, Lagger Grégoire, Storti Ferdinand, Deslarzes Simon, Guillemin Michel, Rossi Michel J, Riediker Michael (2011). Biomarkers of oxidative stress and its association with the urinary reducing capacity in bus maintenance workers. J Occup Med Toxicol.

[30] Chen J, Huang Y, Pan C, Hu C, Chao M (2011). Determination of urinary malondialdehyde by isotope dilution LC-MS/MS with automated solid-phase extraction: a cautionary note on derivatization optimization. Free Radic Biol Med.

[31] Thomas P, Holland N, Bolognesi C, Kirsch-Volders M, Bonassi S, Zeiger E, Knasmueller Siegfried, Fenech Michael (2009). Buccal micronucleus cytome assay. Nat Protoc.

[32] Hopf Nancy B, Bolognesi Claudia, Danuser Brigitta, Wild Pascal (2019). Biological monitoring of workers exposed to carcinogens using the buccal micronucleus approach: A systematic review and meta-analysis. Mutation Research/Reviews in Mutation Research.

[33] Kauffmann F, Annesi-Maesano I, Liard R, Paty E, Faraldo B, Neukirch F, Dizier M H (2002). [Construction and validation of a respiratory epidemiological questionnaire]. Rev Mal Respir.

[34] Martin Remy Aurélie, Wild Pascal (2017). Bivariate Left-Censored Measurements in Biomonitoring: A Bayesian Model for the Determination of Biological Limit Values Based on Occupational Exposure Limits. Ann Work Expo Health.

[35] Bohadana Abraham, Michaely Jean-Pierre, Teculescu Dan, Wild Pascal (2008). Reproducibility of exhaled nitric oxide in smokers and non-smokers: relevance for longitudinal studies. BMC Pulm Med.

[36] Bonassi Stefano, Coskun Erdem, Ceppi Marcello, Lando Cecilia, Bolognesi Claudia, Burgaz Sema, Holland Nina, Kirsh-Volders Micheline, Knasmueller Siegfried, Zeiger Errol, Carnesoltas Deyanira, Cavallo Delia, da Silva Juliana, de Andrade Vanessa M, Demircigil Gonca Cakmak, Domínguez Odio Aníbal, Donmez-Altuntas Hamiyet, Gattas Gilka, Giri Ashok, Giri Sarbani, Gómez-Meda Belinda, Gómez-Arroyo Sandra, Hadjidekova Valeria, Haveric Anja, Kamboj Mala, Kurteshi Kemajl, Martino-Roth Maria Grazia, Montero Montoya Regina, Nersesyan Armen, Pastor-Benito Susana, Favero Salvadori Daisy Maria, Shaposhnikova Alina, Stopper Helga, Thomas Philip, Torres-Bugarín Olivia, Yadav Abhay Singh, Zúñiga González Guillermo, Fenech Michael (2011). The HUman MicroNucleus project on eXfoLiated buccal cells (HUMN(XL)): the role of life-style, host factors, occupational exposures, health status, and assay protocol. Mutat Res.

[37] Sauvain Jean-Jacques, Hohl Magdalena Sánchez Sandoval, Wild Pascal, Pralong Jacques, Riediker Michael (2014). Exhaled breath condensate as a matrix for combustion-based nanoparticle exposure and health effect evaluation. J Aerosol Med Pulm Drug Deliv.

[38] Hulo Sébastien, Chérot-Kornobis Nathalie, Howsam Mike, Crucq Sébastien, de Broucker Virginie, Sobaszek Annie, Edme Jean-Louis (2014). Manganese in exhaled breath condensate: a new marker of exposure to welding fumes. Toxicol Lett.

